# MYCO WELL D-ONE detection of *Ureaplasma* spp. and *Mycoplasma hominis* in sexual health patients in Wales

**DOI:** 10.1007/s10096-020-03993-7

**Published:** 2020-07-28

**Authors:** Daniel J. Morris, Lucy C. Jones, Rebecca L. Davies, Kirsty Sands, Edward Portal, Owen B. Spiller

**Affiliations:** 1 School of Medicine, Division of Infection and Immunity, Department of Medical Microbiology, University Hospital of Wales, Cardiff University, Cardiff, CF14 4XN UK; 2 Department of Integrated Sexual Health, Dewi Sant Hospital, Cwm Taf Morgannwg University Health Board, Pontypridd, CF37 1LB UK

**Keywords:** Ureaplasma, Mycoplasma, Antimicrobial susceptibility, Assay validation, Bacterial load

## Abstract

The genital mycoplasmas are a unique group of inherently antibiotic-resistant sexually transmitted bacteria, often associated with non-gonococcal urethritis and bacterial vaginosis. The MYCO WELL D-ONE is a culture-based assay that aims to detect these organisms whilst concurrently screening them for antibiotic resistance. Urine and/or swabs from 856 informed and consented participants attending Welsh sexual health clinics were subjected to MYCO WELL D-ONE analysis, alongside qPCR and culture titration methodologies to determine sensitivity, specificity, PPV, NPV and accuracy. Resistance was confirmed by CLSI-compliant susceptibility testing and genetic mechanisms determined. The MYCO WELL D-ONE displayed a sensitivity and specificity of 91.98% and 96.44% for the detection of *Ureaplasma* spp., with sensitivity and specificity values of 78.23% and 98.84% for *Mycoplasma hominis*, compared with qPCR. Swabs harboured significantly greater bacterial loads than urine samples for both *Ureaplasma* spp. and *M*. *hominis*. Levofloxacin resistance rates, mediated by Ser83Leu mutation in ParC, for *Ureaplasma* spp. were 0.54%. Tetracycline resistance rates, mediated by *tet*(M), were 0.54% and 2% for *Ureaplasma* spp. and *M*. *hominis*, respectively; sequence analysis of *tet*(M)-positive *Ureaplasma* spp. and *M*. *hominis* strains isolated from a single individual confirmed separate resistance gene origins. The MYCO WELL D-ONE is a sensitive and specific assay for the detection of *Ureaplasma* spp. and *M*. *hominis* in genitourinary medicine samples, facilitating the accurate detection of these organisms within low-technology environments. While good for antibiotic resistance screening, accurate confirmation by MIC determination or molecular methods are required, and more optimally performed on urine samples.

**Electronic supplementary material:**

The online version of this article (10.1007/s10096-020-03993-7) contains supplementary material, which is available to authorized users.

## Introduction

*Ureaplasma* spp. (*Ureaplasma parvum* and *Ureaplasma urealyticum*) and *Mycoplasma hominis* are often colloquially termed the ‘genital mycoplasmas’ (alongside *Mycoplasma genitalium*) due to their isolation from the human urogenital tract. All of the unique class *Mollicutes*, named from the Latin *mollis* (meaning soft) and *cutis* (meaning skin), lack a cell wall [1]. This idiosyncratic physiology, directly related to their reduced genome and subsequent limited biosynthetic capacity results in an inherent resistance to several groups of antibiotics, the β-lactams (including penicillins and cephalosporins), the glycopeptides, sulphonamides and trimethoprim [2, 3]. Consequently, treatment options for these unique bacteria are limited, restricted to four classes of antibiotics: fluoroquinolones, chloramphenicol, tetracyclines and macrolides. Further restrictions apply to pregnant females and neonates as administering all classes besides macrolides are contraindicated. Furthermore, *M*. *hominis* possesses a species-specific resistance to 14- and 15-membered ring macrolides, mediated by a G2057A transition in its 23S rRNA sequence [4], but remain susceptible to 16-membered ring macrolides such as Josamycin.

Though not recognised as equivalent to chlamydia, gonorrhoea or *Mycoplasma genitalium* as pathogens, these innately resistant bacteria are implicated in several urogenital pathologies, with *Ureaplasma* spp. being the only infectious agent in 10–20% of non-gonococcal urethritis (NGU) cases [5], important as currently 45% of NGU are categorised as idiopathic [6]. In females, *Ureaplasma* spp. has been reported as associated with non-specific cervicitis (NSC) [7], endometritis [8] and preterm birth [9]. Whereas *M*. *hominis* is implicated in bacterial vaginosis (BV) [10] and pelvic inflammatory disease (PID) [11]. However, despite mounting evidence associating these organisms with disease, much controversy still surrounds them, owed to their isolation from seemingly healthy asymptomatic populations. However, although *Chlamydia trachomatis* presents as asymptomatic in most women, genital tract chlamydial infections are well accepted as the leading cause of PID, tubal factor infertility and ectopic pregnancy [12]. The dichotomy encompassing their classification as either ‘commensal’ or ‘pathogen’ means the recommendation to routinely screen for and treat these organisms is currently withheld pending more robust, better-designed studies that unequivocally solidify their role in disease [13]. Historically, studies presenting conflicting conclusions on the pathogenicity of *Ureaplasma* spp. and *M. hominis* failed to separate Ureaplasma into the 2 separate species or determine bacterial load, factors shown to influence the pathogenic potential of genital *Mollicutes* infection [14–16]. Consequently, researchers now suggest a risk-based treatment approach toward *Ureaplasma* spp. infection be taken—one considering *Ureaplasma* species, load and known risk factors (age, number of sexual partners etc.)—in cases of symptomatic NGU, devoid of more accepted etiological agents (i.e. chlamydia, gonorrhoea, *M*. *genitalium*, etc.) [17].

Traditional culture-based detection and enumeration methods for these organisms are complicated by their fastidious growth requirements and inability to grow to visual turbidity. Possessing a urea-metabolising enzyme, *Ureaplasma* spp. are unique among mycoplasmas, which are typically characterised by the substrate utilised for ATP generation: arginine or glucose (arginine in the case of *M*. *hominis*) [18]. Culture-based detection techniques employ *Ureaplasma* spp. ability to produce ammonium ions and increase pH through urea hydrolysis. Similarly, the arginine-dihydolase pathway produces ammonium ions and raises pH during the broth culture of *M*. *hominis*. Bespoke media exploit these substrate-specific pathways for detection, visualized through the addition of a pH indicator (typically phenol red) and increased pH from bacterial growth being visualised by a non-turbid yellow to cerise-red colour change in the presence of beta-lactam antibiotics. More sensitive and specific approaches for the detection of these fastidious organisms are available through qPCR, which should be viewed as the ‘gold-standard’ detection method [19] which permits *Ureaplasma* speciation. Though more sensitive, qPCR-based approaches remain uneconomical and impractical for genital *Mollicutes* detection in low-resource laboratories with low to moderate sample volumes, where culture-based assays are better-suited. A key advantage of culture-based approaches is that they provide a viable isolate on which antimicrobial susceptibility testing (AST) can be performed. In commercially available diagnostic assays, this is done simultaneously alongside detection, through supplementing wells containing specific growth medium with breakpoint concentrations of antibiotics. Several commercially available detection kits have been utilised for detection and antimicrobial resistance reporting of *Ureaplasma* spp. and *M*. *hominis*: MYCOFAST revolutioN (ELiTech Diagnostic, France), Mycoplasma Duo and Mycoplasma SIR (Bio-Rad; Watford, U.K), *Mycoplasma* system plus (Liofilchem, Italy), Mycoplasma IST2 (Biomerieux, France). However, these assays fail to utilise CLSI (Clinical Laboratory Standards Institute) set international threshold concentrations of a standardised antibiotic panel, often leading to inappropriate and unvalidated reporting of resistance data [20, 21]. Commercial assays also often fail to take into account the quantification of the bacterial inoculum tested, set by CLSI guidelines as 10^4^–10^5^ colour changing units/mL (CCU/mL), as higher bacterial loads have a demonstrable propensity to produce false positive results [22]. Alongside this, many kits do not separate *Ureaplasma* spp. and *M*. *hominis* coinfection, which complicates interpreting the AST, leading to the over-reporting of macrolide resistance due to *M*. *hominis* intrinsic macrolide resistance [20, 23]. In response, CPM SAS (Rome, Italy) developed the MYCO WELL D-ONE—the first commercially available assay that offers CLSI-compliant antibiotic breakpoints for AST of *Ureaplasma* spp. and *M*. *hominis* separately.

The present investigation was carried out to determine the sensitivity and specificity of the MYCO WELL D-ONE against gold-standard qPCR detection methods alongside culture detection in specialist culture media and CLSI-compliant broth microdilution confirmation of detected antimicrobial resistance.

## Materials and methods

### Participant samples

Urine and/or swabs (urethral, endocervical or high-vaginal) were collected from informed and consented participants—under ethical approval (IRAS 230693)—from October 2017 to October 2018 attending Welsh sexual health walk-in clinics held at Dewi Sant hospital (Pontypridd), Keir Hardie Health Park (Merthyr Tydfil), or Ysbyty Cwm Cynon (Mountain Ash). A total of 983 samples were collected from 856 patients; 526 female patients, of which 122 provided both urine and endocervical swabs; 335 males, of which 5 provided both urine and urethral swabs.

### Sample processing protocol

For qPCR analysis, participant’s urine samples were processed as follows: 2 mL of urine was transferred to a sterile 2 mL Eppendorf tube and centrifuged at 15,000*×g* for 20 min, supernatant removed and pellet frozen at − 86 °C until extraction of genomic DNA using the QIAGEN QIACube automated extraction system (DNeasy Blood & Tissue kit, bacterial pellet protocol with RNase treatment; elution into 100 μL molecular grade water). Participant’s swab samples were processed as follows: Copan Rayon swab samples were either taken by the clinician (endocervical) or self-taken by the participant (high vaginal), the swab was then suspended in 10 mL sterile saline (CPM S.A.S.), 2 mL of the swab suspension was centrifuged and processed for DNA extraction as for urine above. DNA extracts were stored at − 86 °C until batch analysed by quantitative PCR. Concurrently, the MYCO WELL D-ONE assay (purchased from CPM S.A.S., Rome, Italy) was processed as per manufacturer’s instructions. Briefly, 150 μL of swab- (swab resuspended in 10 mL saline) or urine-inoculated saline (200 μL urine added to 10 mL saline) was transferred to each well, topped with two drops of paraffin oil to create an anaerobic environment and stop evaporation and incubated at 37 °C and ambient CO_2_. Additionally, alongside the MYCOWELL D-ONE and qPCR assays, a culture titration assay was implemented to concurrently detect and determine the bacterial load of each swab/urine-inoculated saline sample. The culture titration assay for *Ureaplasma* has been previously described [22] and was modified to include the additional titration of *M*. *hominis* in parallel using *M*. *hominis-*specific medium (MHSM) purchased from CPM SAS (Rome, Italy). Briefly, titration and breakpoint screening in antibiotics were set up in sterile 96-well flat-bottomed microtitre plates receiving 10 μL of sample-inoculated sterile saline containing 90 μL of *Ureaplasma* or *M*. *hominis-*specific medium prior to 1:10 dilution series in the same medium to a final 10^−7^dilution. Uninoculated medium was included in parallel and acted as a negative control for each serial dilution; the plates were then sealed with transparent adhesive sealing tape (Elkay: Basingstoke, United Kingdom), and were incubated at 37 °C and ambient CO_2_. Alongside this culture titration methodology, 20 μL of undiluted urine was pipetted into 180 μL USM and MHSM and incubated at 37 °C and read at various intervals up to 72 h post-incubation; this latter method was included because 10 μL of 1:500 diluted urine in sterile saline was 15-fold less than the amount inoculated into the MYCO WELL D-ONE and has an overall limit of detection of 10^3^ CCU per mL. Finally, 5 μL of inoculated sterile saline solution was pipetted directly onto the surface of US1 agar (Mycoplasma Experience) and incubated at 37 °C and ambient CO_2_. Positives were determined through the presence of a classic ‘fried-egg’ morphology under light microscopy for *M*. *hominis*, and red pH colour change in the media with tiny granular *Ureaplasma* colonies.

### Molecular analysis

BioRad Laboratories (Watford, UK) CFX96 Touch Real-Time PCR thermocycler and BioRad Maestro software was used to run the assays. SsoAdvanced Universal Probes Supermix for hydrolysis probes (BioRad Laboratories) and 96-well plates and optical clear adhesive seals (BioRad Laboratories) were used. Total volume of each reaction was 20 μL, comprised of the following: 10 μL 2x mastermix, 0.25 pmol/μL *Ureaplasma parvum* forward primer, 0.25 pmol/μL *Ureaplasma parvum* reverse primer, 0.01 pmol/μL *Ureaplasma parvum* probe; 0.25 pmol/μL *Ureaplasma urealyticum* forward primer, 0.25 pmol/μL *Ureaplasma urealyticum* reverse primer, 0.01 pmol/μL *Ureaplasma urealyticum* probe; 0.25 pmol/μL *Mycoplasma hominis* forward primer, 0.25 pmol/μL *Mycoplasma hominis* reverse primer, 0.01 pmol/μL *Mycoplasma hominis* probe, with the remainder molecular grade water. Genomic copy equivalents were determined against a 6-point standard curve (10^6^–10^1^ copies) of diluted plasmid containing concatamers of the primer and probe targets separated by 30 bp intervening sequences (synthesized by GenScript) and accurately quantified by Life Technologies Qubit fluorometer DNA quantification system. Primers and Taqman hydrolysis probes for *Ureaplasma parvum* and *Ureaplasma urealyticum* were previously clinically validated [24] as were primers and Taqman hydrolysis probes for *Mycoplasma* hominis [25]. Bio-Rad CFX96 cycling conditions were as follows: initial activation and denaturation 95 °C for 5 min, next 45 cycles of 95 °C for 15 s and 60 °C for 15 s. Readings were acquired in between cycles on channels FAM, HEX, ROX and CY5. Data was analysed on Bio-Rad CFX Maestro software. Presence of amplification inhibitors were assessed using QIAGEN Quantinova as per manufacturer’s instructions. Primers and probes are listed in Supplementary Table 1.

### CLSI-compliant break-point antimicrobial susceptibility testing

Analysis of putative resistant isolates was conducted in accordance with CLSI M43A(21) using a previously published methodology for breakpoint analysis for *Ureaplasma* spp. isolates [22]. The method was modified to include additional antibiotics, alongside utilising MHSM for the breakpoint screening of *M*. *hominis* isolates. Isolates were screened against the CLSI guideline concentrations of antibiotic. Isolates were only screened against antibiotics if they had shown supposed resistance to that antibiotic on the MYCO WELL D-ONE assay. All antibiotics were purchased from Sigma-Aldrich (Dorset, UK) and supplied as or reconstituted to 1 mg/mL stock solutions, prior to further dilution in the appropriate media (USM/MHSM) to achieve CLSI-compliant concentrations for AST.

### Whole genome sequencing

All isolates confirmed to be antibiotic resistant by CLSI-compliant methods were scaled up to 500 mL culture, pelleted at 13,000*×g* for 3 h and DNA extracted using the QIAcube and QIAGEN reagents for Gram-negative protocol described above. Following extraction of DNA, Qubit 4.0 (Life Technologies) fluorometric analysis verified a sufficient concentration of DNA was isolated (1–8 ng/μL). Briefly, whole genome sequencing was performed using a Nextera XT library preparation and sequenced with a V3 chemistry on an Illumina MiSeq platform. The bioinformatics pipeline was comprised of 3 main processes; (1) QC pipeline to validate and trim the raw sequence reads: FastQC and Trimgalore [26, 27]; (2) whole genome assembly and mapping: Flash, SPAdes, BWA, pilon and quast [28–32]; (3) whole genome annotation and profiling of genetic determinants using a combination of available software (using both fastq and de novo assembled reads): prokka, NCBI BLAST, kmerfinder, CARD, srst2, ARG-ANNOT and VFDB [33–39]. Assembled contigs were further analysed utilising Geneious sequence analysis software (BioMatters Ltd., New Zealand) and aligned and assessed against antibiotic susceptible reference sequences for the identification of mobile genetic elements or point mutations.

### Statistical analysis

All statistical analyses were performed using GraphPad Prism version 7 (GraphPad Software). Significance was only attributed to comparisons where *P* value < 0.05.

## Results

### Assay sensitivity and specificity

The multiplex qPCR implemented for the detection of *Ureaplasma* spp. and *M*. *hominis* infections was utilised as the ‘gold standard’ reference methodology to determine the sensitivity and specificity of the MYCO WELL D-ONE assay detecting these organisms (Tables 1 and 2). For the detection of *Ureaplasma* spp. from 983 samples, 413 qPCR positive samples, 515 qPCR negative samples, 19 false positive (qPCR negative, MYCO WELL D-ONE positive) samples and 26 false negative (qPCR positive, MYCO WELL D-ONE negative) samples were identified, when comparing MYCO WELL D-ONE data with that of qPCR. Utilising these data, the sensitivity and specificity for the detection of *Ureaplasma* infection for the MYCO WELL D-ONE is calculated to be 91.98% and 96.44%, respectively. The positive predictive value (PPV) and negative predictive value (NPV) of the MYCO WELL D-ONE to determine *Ureaplasma* infection was 95.6% and 93.47%, respectively, with an accuracy of 94.4%. For the detection of *M*. *hominis*, a total of 97 true positive samples, 849 true negative samples, 10 false positive samples and 27 false negative samples were identified when comparing MYCO WELL D-ONE data with that of qPCR. The sensitivity and specificity for the detection of *M*. *hominis* infection for the MYCO WELL D-ONE is calculated to be 78.23% and 98.84%, respectively. The positive predictive value (PPV) and negative predictive value (NPV) of the MYCO WELL D-ONE to determine *Mycoplasma hominis* infection was 90.65% and 96.92%, respectively, with an accuracy of 96.2%.

**Table 1:** Sensitivity, specificity, positive predictive value, negative predictive value and accuracy for MYCO WELL D-ONE detection of *Ureaplasma* spp.

Detection of *Ureaplasma* spp.	Total sample number	Sensitivity	Specificity	Positive predictive value	Negative predictive value	Accuracy
Overall	983	91.98%	96.44%	95.60%	93.47%	94.40%
Swab	297	92.05%	93.39%	98.29%	88.98%	92.59%
Urine	686	91.94%	99.27%	98.82%	94.91%	96.36%
Male	335	85.71%	99.62%	98.36%	96.35%	96.72%
Female	648	93.14%	93.31%	95.15%	90.61%	93.21%

Relative to qPCR detection of *Ureaplasma* spp. infection across sample types and genders. The MYCO WELL D-ONE was most sensitive for the detection of *Ureaplasma* spp. infections in females (93.14%), particularly for swab samples (92.05%). Specificity and accuracy were highest for male (99.62% and 96.72%, respectively) and urine samples (99.29% and 96.36%, respectively)

**Table 2:** Sensitivity, specificity, positive predictive value, negative predictive value and accuracy for the MYCO WELL D-ONE detection of *Mycoplasma hominis*

Detection of *M*. *hominis*	Total sample number	Sensitivity	Specificity	Positive predictive value	Negative predictive value	Accuracy
Overall	983	78.23%	98.84%	90.65%	96.92%	96.24%
Swab	297	78.57%	97.93%	89.80%	95.16%	94.28%
Urine	686	77.46%	99.35%	93.22%	97.45%	97.09%
Male	335	92.86%	99.69%	92.86%	99.69%	99.40%
Female	648	76.36%	98.33%	90.32%	95.32%	94.60%

Relative to qPCR detection of *M*. *hominis* infection across sample types and genders. The MYCO WELL D-ONE was most sensitive for the detection of *M*. *hominis* infections in males (92.86%). Specificity and accuracy were highest for male (99.69% and 99.40%, respectively) and urine samples (99.35% and 97.09%, respectively)

### Antimicrobial screening accuracy

**Fig. 1:**
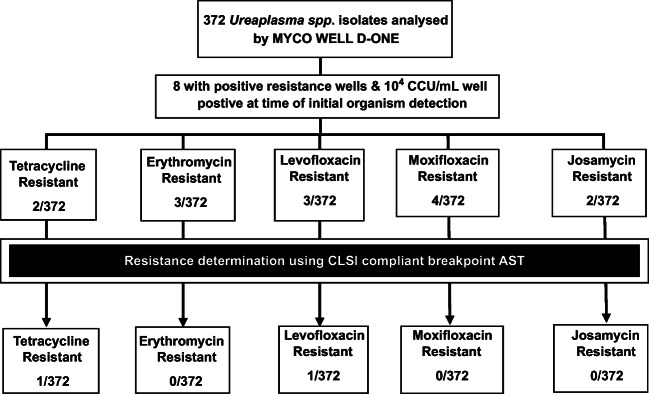
Flow chart displaying CLSI-compliant AST of *Ureaplasma* spp. presumptively identified as resistant by MYCO WELL D-ONE. The MYCO WELL D-ONE was not found to significantly over-report resistance (*p* > 0.05; Fisher’s exact test). For isolates that were identified by the assay as being resistant (without additional incubation post-organism detection) and at a bacterial load of 10^4^ CCU/mL, one isolate was confirmed as resistant to levofloxacin and one isolate to tetracycline. Further resistant *Ureaplasma* spp. isolates were found (1 levofloxacin and 1 tetracycline) though are omitted from this chart as they were identified as ≥10^5^ CCU/mL by the initial assay screening

All samples displaying a positive result in the highest concentration of antibiotic (set at the CLSI determined breakpoint concentrations) were further investigated for antimicrobial resistance utilising the methodology outlined in Beeton et al. [22]. The guidance included with the MYCO WELL D-ONE assay stipulates that if the ≥ 10^5^ well is positive, any positivity in resistance determining wells is invalid and is to be confirmed by other means. In total, 106 *Ureaplasma* spp. isolates were culture purified through serial dilution in specialist media and screened for antibiotic resistance against one or more antibiotic, due to positivity displayed in AST wells of the MYCO WELL D-ONE. However, these represent all strains that showed colour change for any antibiotic at 72 h, and adherence to manufacturer’s guidelines (only consider those that do not show colour change for the ≥10^5^ bacterial load and disregard antibiotic resistance that emerges at timepoints later than the initial positive Mollicute identification) greatly reduced the number of false positives identified. Uncorrected putative resistance included 69 for erythromycin, 25 for josamycin, 30 for tetracycline, 32 for levofloxacin and 23 for moxifloxacin. Of those tested, 94 were excluded due to a positive ≥ 10^5^ MYCO WELL D-ONE well. By manufacturer’s guidelines, these samples would have required confirmation following dilution and retesting or by alternative method. The 12 remaining isolates were predicted to have been tested at a bacterial load of 10^4^ CCU/mL with various combinations of positive resistance wells; however, antimicrobial resistance was only seen for 4 of these after 12–24 h additional incubation and must also be excluded (Fig. 1). Isolates were screened against one or more antibiotic; 3 against erythromycin, 2 against josamycin, 2 against tetracycline, 3 against levofloxacin and 4 against moxifloxacin. Of these 8 isolates determined to be resistant by MYCOWELL D-ONE, two (1 levofloxacin-resistant and 1 tetracycline-resistant) were confirmed to be resistant when subjected to CLSI compliant AST. A further 2 resistant isolates (1 levofloxacin-resistant and 1 tetracycline-resistant) were discovered following screening of isolates that possessed a positive > 10^5^CCU/mL well, indicating that resistance confirmation should be determined by other means or retested following dilution. In total, 2 levofloxacin-resistant strains (isolates DF99 and KF86) and 2 tetracycline-resistant strains (isolates DF145 and DF28) were identified to be truly resistant. These isolates were scaled up and analysed for whole genome sequencing.

**Fig. 2:**
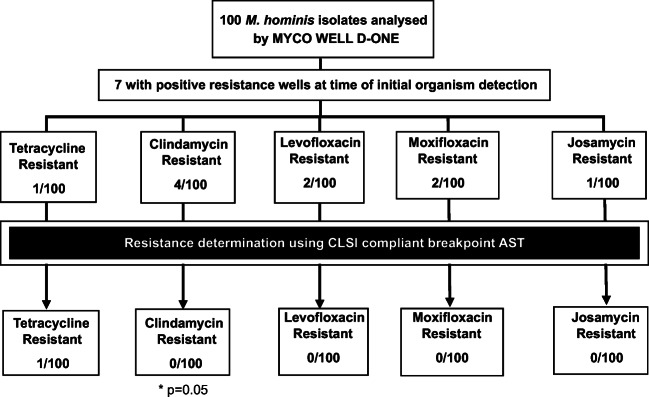
Flow chart displaying CLSI-compliant AST of *M*. *hominis* presumptively identified as resistant by MYCO WELL D-ONE. The MYCO WELL D-ONE over-reported clindamycin resistance (*p* = 0.05; Fisher’s exact test). One further tetracycline-resistant *M*. *hominis* isolate was found although it was missed by the initial assay screening

For *M*. *hominis*, 14 isolates were identified as resistant on the MYCO WELL D-ONE assay (Fig. 2); however, the assay does not indicate bacterial load for this species to determine if they are compliant with CLSI guidelines. However, for 7 of these isolates, antibiotic resistance was only detected after 13–28 h after initial organism’s identification and were therefore excluded as directed by manufacturer’s guidelines. Therefore, the remaining putative resistant isolates were culture purified and screened against one or more antibiotics; 4 against clindamycin, 1 against josamycin, 1 against tetracycline, 2 against levofloxacin and 2 against moxifloxacin. However, only a single *M*. *hominis* isolate was found to be tetracycline resistant (isolate DF28) following comprehensive confirmatory testing. AST analysis of the remaining *M*. *hominis* isolates identified a further tetracycline-resistant isolate (KM14) missed by the MYCO WELL D-ONE assay (Fig. 2).

### Mechanisms of antimicrobial resistance determination

**Fig. 3:**
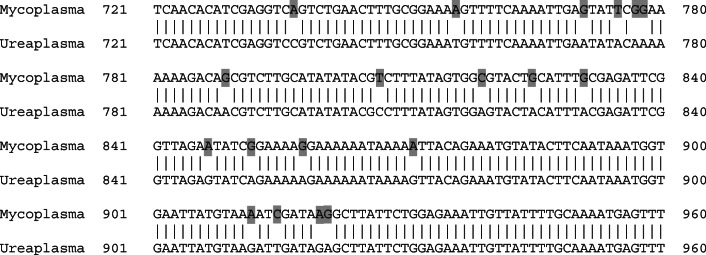
Nucleotide alignment of the *tet*(M) gene found in the *U*. *parvum* and *M*. *hominis* strains, both co-incidentally isolated from the same patient. Shown here are nineteen of the twenty-four mismatches in nucleotide identity were clustered in a 180 nucleotide stretch in the middle of the 1920 nucleotide open reading frame, confirming different origins for these genes and not transfer between these two co-infecting species

Whole genome sequencing of resistant isolates revealed the genetic elements and somatic mutations conferring the antibiotic resistance to the isolated organism (Table 3). Both *Ureaplasma* spp. isolated and determined to be levofloxacin resistant (MIC = 4 mg/L) possessed the amino acid substitution Ser83Leu (serine to leucine substitution) in the *parC* gene of the quinolone resistance determining region (QRDR). The tetracycline-resistant *Ureaplasma* spp. isolated both possessed the Tn916 *tet*(*M*) conjugated transposon as did the two tetracycline-resistant *M*. *hominis* isolates. The patient identified as DF28 yielded both a tet(M)-positive *U*. *parvum* strain and a tet(M)-positive *M*. *hominis* strain; however, direct comparison of the genes found a difference of 24/1920 nucleotides (19 of which were clustered in close proximity; Fig. 3), indicating completely separate origins for these strains coincidental in their infection of the same patient.

**Table 3:** Details of resistant isolates

Sample number	Antibiotic	MIC (μg/mL)	Resistance mechanism	Species
DF99	Levofloxacin	4	Ser83Leu ParC mutation	*U*. *parvum*
KF86	Levofloxacin	4	Ser83Leu ParC mutation	*U*. *parvum*
DF28	Tetracycline	128	Tn916 *tet*(*M*)^a^	*U*. *parvum*
DF145	Tetracycline	32	Tn916 *tet*(*M*)	*U*. *parvum*
DF28	Tetracycline	64	Tn916 *tet*(*M*)^a^	*M*. *hominis*
KM14	Tetracycline	64	Tn916 *tet*(*M*)	*M*. *hominis*

MIC determined under CLSI-compliant conditions are given alongside the underlying genetic mechanism of resistance identified by whole genome sequencing analysis

^a^*tet*(*M*) gene sequence from isolated *U*. *parvum* and *M*. *hominis* strains differed by 24/1920 nucleotides and therefore originated from different sources

**Fig. 4:**
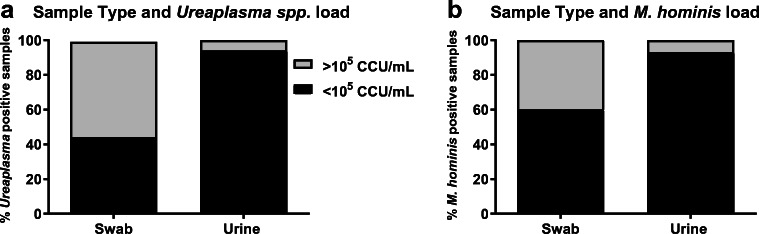
Bacterial loads for *Ureaplasma* spp. (**a**) or *M*. *hominis* (**b**) in swab or urine samples. Bacterial loads from urine samples were more frequently amenable for AST, based on CLSI guidelines, for < 10^5^ CCU/ml (black fraction of the bar). **a** For *Ureaplasma*-positive urine samples 5.9% possessed a load > 10^5^ CCU/mL (grey fraction of the bar), whereas for *Ureaplasma*-positive swab samples, 55.9% possessed a load > 10^5^ CCU/mL. The prevalence of a *Ureaplasma* load of > 10^5^ CCU/mL captured on swab samples was significantly higher than that of urine samples, for Ureaplasma positive patients (*p* ≤ 0.0001; Fisher’s exact test). **b** For *M*. *hominis*-positive urine samples, 7% possessed a load > 10^5^ CCU/mL. Whereas for *M*. *hominis*-positive swab samples, 40% possessed a load > 10^5^ CCU/mL. The prevalence of a *M*. *hominis* load of > 10^5^ CCU/mL captured on swab samples was significantly higher than that of urine samples, for *M*. *hominis* positive patients (*p* = 0.0004; Fisher’s exact test)

### Bacterial load comparison for sample type

It was observed that analysis of *Ureaplasma* spp. samples from swabs routinely were identified as having > 10^5^CCU/mL bacterial loads by MYCO WELL D-ONE identification wells. Accurate bacterial load quantification by titration in selective media was used to compare data for different sample types and genders for both and *Ureaplasma* spp. and *M*. *hominis*. Given that these dilutions were made from samples suspended in 10 mL of sterile saline initially, the limit of detection for the culture titration methodology would be 10^3^CCU/mL. Therefore, where this method failed to grow isolates, that were detected in parallel either via the single well inoculation of undiluted urine or detection of colonies on inoculated US1 agar, these were recorded to have a bacterial load of < 10^3^CCU/mL for analysis. *Ureaplasma* spp. were detected in 422 samples: 168 swab samples and 254 urine samples; and for *M*. *hominis*, 91 samples were culture-positive; 48 swab samples and 43 urine samples. Positive samples were divided into those greater, and those less, than 10^5^CCU/mL (CLSI set limit for accurate AST) and further sub-categorised by sample type. For *Ureaplasma*-positive urine samples, 5.9% (15/254) were > 10^5^ CCU/mL, with 94.1% of samples having *Ureaplasma* load of < 10^5^CCU/mL. While for swab samples, 55.9% of *Ureaplasma* positives had a bacterial load of > 10^5^ CCU/mL, with the remainder of positives (44.1%) having *Ureaplasma* load of < 10^5^CCU/mL (Fig. 4a). The prevalence of *Ureaplasma* load of > 10^5^CCU/mL captured on swab samples is significantly higher than that of urine samples, for *Ureaplasma-*positive patients (*p* < 0.0001; Fisher’s exact test). Analysis of *M*. *hominis*-positive samples (Fig. 4b) similarly revealed swabs possess greater bacterial loads than *M*. *hominis*-positive urine samples. *M*. *hominis*-positive swab samples with a bacterial load of > 10^5^CCU/mL accounted for 40% of total swab-positives (19/48); 60% of swab-positives had a *M*. *hominis* load of < 10^5^CCU/mL (29/48). Whereas 93% of *M*. *hominis*-positive urine samples had a bacterial load < 10^5^ CCU/mL (40/43) with 7% of positive urines having a *M*. *hominis* load of > 10^5^CCU/mL. Fisher’s exact test determined that *M*. *hominis*-positive swab samples have a statistically significant greater incidence of bacterial loads exceeding 10^5^CCU/mL, when compared with *M*. *hominis* loads of urine-positive samples (*p* = 0.0004). Therefore, these assays are much better suited to urine samples and direct AST evaluation may not be possible on resuspended swabs without modification of the protocol to include additional dilution.

**Fig. 5:**
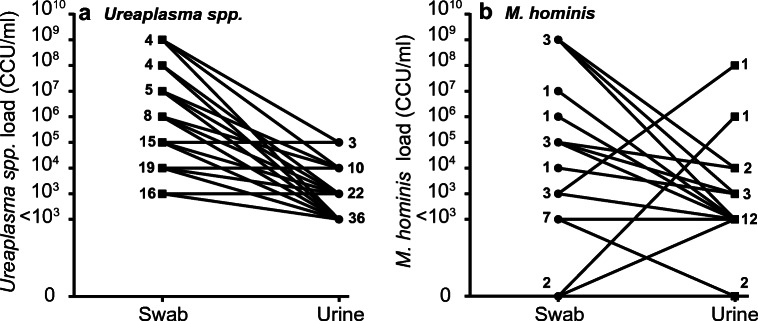
Bacterial load comparison for matched *Ureaplasma*-positive (**a**) or *M. hominis*-positive (**b**) patient samples. **a** All paired *Ureaplasma*-positive samples possessed bacterial loads equal to or greater their urine counterparts. Statistical analysis revealed swabs to possess significantly greater *Ureaplasma* loads than urines (*p* = 0.0255; paired *t* test). **b** 76.2% of paired *M*. *hominis*-positive samples possessed bacterial loads equal to or greater their urine counterparts. However, 2 urine samples detected *M*. *hominis* where swab samples did not and 2 swab samples detected *M*. *hominis* in patients with negative culture results from urine. A universal trend of lower bacterial load in urine compared to matched swabs was not always seen for this organism. Statistical analysis of matched *M*. *hominis*-positive samples failed to achieve significance (*p* = 0.0939; paired *t* test)

In total, 127 patients provided paired swab and urine samples, comprised of the following: 122 endocervical swab-urine pairings from female patients and 5 urethral swab-urine pairings from male patients, 71 patients were positive for *Ureaplasma* spp. and 21 patients *M*. *hominis*-positive by culture titration. The limit of detection for the culture titration method was 10^3^CCU/mL. Therefore, as above, detection by culture methods run in parallel not subjected to these limitations are listed as < 10^3^CCU/mL and where no organism was detected (*M*. *hominis* only) the matching negative culture is shown as 0 (Fig. 5). For *Ureaplasma* spp. (Fig. 5a), in 38% of cases, swabs had bacterial loads equal to or 10 times greater than their urine counterparts. Whereas bacterial loads between 100 and 1000 times higher in swab samples, compared with their respective urines, were observed in 43.7% of *Ureaplasma*-positive samples. Additionally, swabs with *Ureaplasma* loads between 10^3^ and 10^5^ and 10^6^–10^7^ times greater than their urine counterpart samples were observed in 15.5% and 2.8% of *Ureaplasma*-positive patients, respectively. A paired *t* test between paired urine and swab samples displays swabs have significantly higher bacterial loads than their respective urine samples (*p* = 0.0255). For *M*. *homini*s-positive paired patient samples (Fig. 5b), 76.2% (16/21) possessed swab-positive cultures with bacterial loads equal to, or greater than, their respective urine samples. For 9.5% (2/21) of *M*. *hominis*-positive patients with paired samples, *M*. *hominis* could only be recovered from urine samples and was undetectable in the complementary swab sample. Conversely, for a further 9.5% (2/21) of *M*. *hominis*-positive patients with paired samples, *M*. *hominis* was undetectable in the urine samples, but was recovered from the complementary swab samples. The remaining sample (1/21), accounting for 4.75% of total *M*. *hominis*-positive paired samples, possessed a urine sample with a greater bacterial load than its swab counterpart. Swab samples had *M*. *hominis* loads equal to, or 10 times higher, in 42.9% of cases (9/21). Swab samples with 100–1000 times higher, and swab samples with 10^4^–10^5^ times higher *M*. *hominis* loads accounted for 23.8% and 14.3% of paired samples, respectively. Additionally, 9.5% of paired swab samples had bacterial loads 10^6^–10^9^ times higher, than their respective urine samples. However, in two instances, both comprising 4.5% each of the total *M*. *hominis*-positive paired samples, urine samples had a higher bacterial load than their swab counterparts, one with a bacterial load 100 times greater and the other with a load 10^6^ times higher. Finally, though swab samples more frequently had greater bacterial loads than their respective urine samples, insufficient matched samples were available for these differences to achieve a statistical difference by a paired *t* test (*p* = 0.0939).

### Time to detection from assay inoculation

**Fig. 6:**
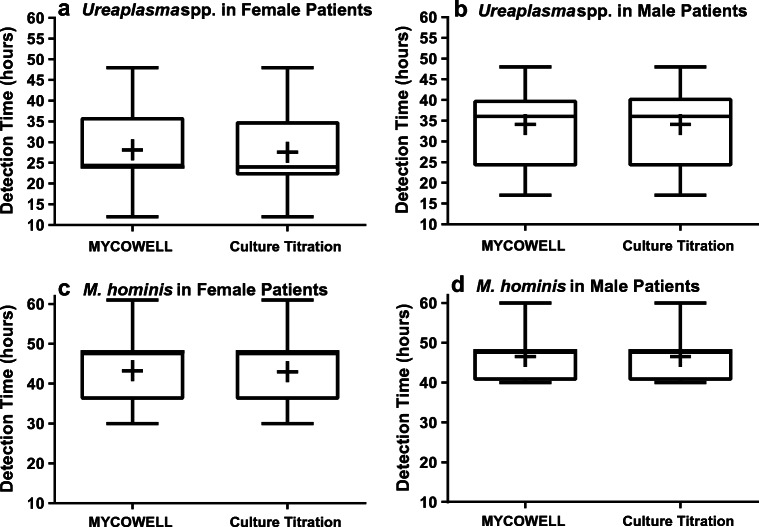
Bar and whisker graphs displaying the min-max detection times for *Ureaplasma* spp. and *M*. *hominis*. Samples from males and females are examined separately comparing the MYCO WELL D-ONE assay to culture titration assay in specialist media. Mean values are represented by + symbol. Time to detection of *Ureaplasma* spp. in female samples was 28.14 h (culture titration) and 27.57 (MYCO WELL D-ONE) (**a**) and 34.09 h in males was for both assays (**b**). Time to detection of *M*. *hominis* in females was 43.26 and 43 h for MYCO WELL D-ONE and culture titration, respectively (**c**), and 46.25 h in male samples (**d**). There was no significant difference in time to detection between assays

A limitation of all non-molecular-based detection and AST testing assays is the incubation time. Therefore, we analysed time to first detection for MYCO WELL D-ONE, alongside the culture titration methodology, was also recorded following incubation (Fig. 6). Plates were inoculated immediately following receipt of the sample at the hospital in which they were collected and placed in an incubator in 3 h or less. Samples were incubated overnight and read at the earliest convenience the next day and then at regular intervals until 72 h. For the culture titration assay, the mean detection time for *Ureaplasma* spp. in female patient samples was 28.14 h post-incubation. Whereas for the MYCO WELL D-ONE, the mean time to detection for *Ureaplasma* spp. in female patients was 27.57 h post-incubation. The mean detection time for *Ureaplasma* spp. in male patient samples was 34.09 h for both assays. Statistical analysis (ANOVA followed by post-hoc analysis using Bonferroni correction) found no difference between methods within genders, but that *Ureaplasma* spp. was identified significantly faster for female patients (*p* < 0.001) than for male patients by either method, and significance was removed if swab samples were excluded from the analysis. For *M*. *hominis*, the mean detection time in female patient samples was 43.26 and 43.00 h for the MYCO WELL D-ONE and culture titration assay, respectively. Whereas for the detection of *M*. *hominis* in male samples, the mean time to a positive result was 46.54 h for both assays. Statistical analysis found no difference for detection time between genders or methodologies, but that *Ureaplasma* spp. was detected a full day faster than *M*. *hominis* by any culture method (*p* < 0.0001).

## Discussion

As the body of evidence associating *Ureaplasma* spp. and *M*. *hominis* with urogenital pathologies continues to grow, tools capable of accurately detecting these organisms—particularly in the low-resource environments of genitourinary medicine clinics—have the potential to offer clinicians a more comprehensive view of the microbial status of symptomatic patients in the absence of more traditional sexual health pathogens (chlamydia, gonorrhoea, *M*. *genitalium*, etc.). Additionally, the inherent antibiotic-resistant nature of these organisms increases the risk of the empirical treatment approaches employed to treat symptomatic non-gonococcal urogenital infections failing. Therefore, systems capable of reliably detecting and concurrently screening these organisms against a panel of commonly prescribed antimicrobials will be valuable in instances to perform proper epidemiological evaluations of the pathogenic potential of these organisms. Additionally, the added information provided by these assays will allow screening for resistance to the subset of antimicrobials that are effective to treat these infections.

For the detection of *Ureaplasma* spp., the MYCO WELL D-ONE has overall sensitivity and specificity of 91.98% and 96.44%, compared with multiplex qPCR. This is consistent with a publication comparing PCR with Mycotest (Bioprepare), showing sensitivity and specificity of 92.4% and 93.8%, respectively [40]. However, wide-ranging sensitivities and specificities for culture-based *Ureaplasma* detection methods versus PCR have been published. For example, the sensitivity of the Mycoplasma IST2 (Biomerieux) compared with a commercially available PCR (Anyplex II; Seegene) was only 44.9%, with a specificity of 87.9% [41]. A previous publication by Favalli et al. has compared the detection of the MYCO WELL D-ONE to the commercial detection kit Anyplex II [42]. These authors were able to evaluate the extended capacity of the kit to detect non-*Mollicutes* and found excellent concordance in detecting 33/39 *Gardnerella vaginalis* and 3/3 *Trichomonas vaginalis* infections. They found slightly lower *Mycoplasma hominis* values (sensitivity 71.4%; sensitivity 90.7%) and significantly lower values for *Ureaplasma* spp. (48.98% and 93.4% for sensitivity and specificity respectively, compared to ours at 91.98% and 96.44%). These relatively low values were due to 4 positives on MYCO WELL D-ONE not detected by Anyplex II, and demonstrates the limitations of a small sample number (*N* = 110) and comparison to only one detection method that was neither quantitative nor regarded as a clinical diagnostic gold standard. We adopted a novel method of confirming colour changed wells by direct subculture and qPCR, which routinely supported the highly sensitive detection of the MYCO WELL D-ONE when load was too low to be detected by our other methods. This highlights the importance of confirming positive cultures with molecular methods to properly categorise ostensibly false positive culture results. For the detection of *M*. *hominis*, the overall sensitivity and specificity was 78.23% and 98.84%, respectively. As a limitation to all molecular detection methods, Favalli et al. were also unable to evaluate the antimicrobial susceptibility screening function of the MYCO WELL D-ONE assay. When comparing PCR with culture, Petrikkos et al. found a 29.6% reduction in detection sensitivity between *Ureaplasma* spp. and *M*. *hominis* with sensitivity declining from 92.4% for *Ureaplasma* spp. to 62.8% for *M*. *hominis*. Similarly, the sensitivity of the MYCO WELL D-ONE declined by 13.75%, from 91.98% for the detection of *Ureaplasma* spp. to 78.23% for the detection of *M*. *hominis*. Petrikkos and colleagues also reported that the specificity for *M*. *hominis* detection remained in relatively high concordance with molecular methods, at 98.8%. Comparable results are presented here with *M*. *hominis* specificity being calculated to be 98.84%. Further support for the notion of culture being less sensitive for the detection of *M*. *hominis*, than it is for the detection of *Ureaplasma* spp., is offered by Abele-Horn et al.’s sensitivity values for *Ureaplasma* spp. and *M*. *hominis* detection at 91% and 84%, respectively [43]. The disparity between sensitivity for the culture-based detection of *Ureaplasma* spp. and *M*. *hominis* has not been explained elsewhere, though it has been determined that *M*. *hominis* are harder to recover by culture-based means than *Ureaplasma* spp.; recovery rate of *Ureaplasma* spp. is 95%, compared with a recovery rate of 60% for *M*. *hominis* [44]. So, it may simply be that through using culture-based assays, *M*. *hominis* is harder to recover than *Ureaplasma* spp. and is perhaps an indication of their more fastidious nature. Across all sample types, the specificity of *M*. *hominis* detection by the MYCO WELL D-ONE was consistently > 97%, demonstrating that the assay had an exceptionally low false positive rate.

The performance of the semi-quantitative well for *Ureaplasma* spp. was unable to be assessed, given an error in our study design. The primary aim of this well is to quantify the *Ureaplasma* spp. inoculum as CLSI-compliant or not (≤ 10^4^ CCU/mL) to minimise the identification false resistance, as it has been previously demonstrated that a failure to correctly quantify *Ureaplasma* concentrations led to an over-reporting of resistance [22]. Unfortunately, we only recorded the results for these wells at 72 h and were unable to retrospectively re-analyse the data as we had done for the antibiotic susceptibility data where time to detection and time of antibiotic well readings were separately recorded. Given that we did not record the times for the *Ureaplasma* spp. bacterial load wells, it would be unfair to analyse the accuracy of these results against definitive methods. We tested whether prolonged incubation led to artificial errors by re-examining 25 *Ureaplasma* isolates at > 10^5^ CCU and < 10^5^ CCU and found that readings at first colour change (16–18 h) were completely accurate for both antimicrobial resistance and bacterial load, but over-estimate for bacterial load occurred for 8 isolates at < 10^5^ CCU and false antimicrobial resistance was found for 3 isolates at 72 h as opposed to accurate readings at first organism identification (16–24 h) (data not shown). Therefore, it is critical that these wells not be allowed to over-incubate.

Levofloxacin resistance for *Ureaplasma* spp. was 0.54%, considerably lower than rates published in other studies. In Minnesota, 250 clinically isolated *Ureaplasma* spp. subjected to MIC testing determined a levofloxacin resistance rate of 6.4% and 5.2% for *U*. *parvum* and *U*. *urealyticum*, respectively [45]. Molecular characterisation of the isolates in the Fernandez et al. study revealed 93% of levofloxacin-resistant *Ureaplasma* spp. to possess the *parC* quinolone-associated resistance mutation, Ser83Leu. Similarly, sequencing of the two levofloxacin-resistant isolates identified here confirmed a Ser83Leu mutation in the *parC* gene. This Ser83Leu *parC* gene mutation is the most prevalent levofloxacin resistance mechanism identified in *Ureaplasma* spp. isolates, accounting for up to 87% of fluoroquinolone resistance [45–48]. Though comparatively low, the levofloxacin resistance rate reported here is not unusual, being concordant in US and French studies: 1.4% and 1.2%, respectively [49, 50]. Conversely, Song et al. reported levofloxacin resistance for *Ureaplasma* spp. in China to be 75% and the most common mechanism of resistance to be mostly conferred by the Ser83Leu *parC* mutation [51]. They attributed the high resistance rates to widespread and general use of fluoroquinolones in China. Beeton et al. determined 2/130 isolates to have relative resistance to ciprofloxacin in samples from England in Wales, but to have an MIC = 2 mg/L for levofloxacin and therefore considered to be below the breakpoint, despite both having the Ser93Leu *parC* mutation [52]. This coupled with our equivalent findings of 2/424 isolates having intermediate resistance (MIC = 4 mg/L levofloxacin) confirm extremely low fluoroquinolone resistance for *Ureaplasma* spp. in Wales.

Tetracycline resistance in *Ureaplasma* spp. was 0.54%, also particularly low. Molecular characterisation of the isolates revealed that both harboured the Tn916 *tet*(*M*) conjugated transposon, the only identified mechanism by which *Ureaplasma* spp. possess tetracycline resistance [53]. Tetracycline resistance rates for *Ureaplasma* spp. typically range between 0 and 14% globally: 0%, Croatia [54]; 1%, China [55]; 2%, Italy [56]; 6%, Hungary [57]; 14%, Turkey [58]. Though tetracycline resistance rates as high as 34% have been published following CLSI-compliant AST of *Ureaplasma* spp. isolates in the USA [46]. However, more recently, Valentine-King and Brown published a 1.4% tetracycline resistance rate for *Ureaplasma* spp. in Florida, USA [49]. Comparatively, screening for tetracycline resistance in *Ureaplasma* spp. populations in Wales and England has consistently reported rates of 2% [22, 52]. The findings of this study show that *tet*(*M*) prevalence in *Ureaplasma* spp. populations throughout Wales have not appreciably increased. Likewise, a 6-year analysis of *Ureaplasma* spp. isolates in France over a 6-year period (2010–2015) did not note any increase in *tet*(*M*) prevalence, ~ 7.5% [50]. But, previous French data (1999–2002) determined *Ureaplasma* spp. tetracycline resistance rates to be at 2.2% [59]. Therefore, whilst no significant change was seen in the most recent 6-year assessment, there has been an overall rise in tetracycline resistance over the last 20 years. This highlights the need for continued antibiotic resistance surveillance for *Ureaplasma* spp. to track such changes in geographically distinct locations, especially as tetracycline is prescribed as a first-line therapy in the treatment of adult urogenital *Ureaplasma* spp. infection. For *M*. *hominis*, 2 tetracycline-resistant isolates were identified giving a relatively low resistance rate of 2%. However, reported resistance for *M*. *hominis* varies greatly, ranging from 0 to 100% [60–62]. Though typically, resistance rates are situated between 10 and 40% of *M*. *hominis*, globally [63, 64]. Both isolates determined to be tetracycline-resistant harboured the Tn916 *tet*(*M*) gene. Like *Ureaplasma* spp., this is the most described resistance mechanism for tetracycline resistance in *M*. *hominis*. However, it is important to note that while a *tet*(*M*)-positive tetracycline-resistant *Ureaplasma parvum* and *M*. *hominis* were both isolated from the same patient (DF28), sequence identity of the genes was only 1896/1920 bp, indicating they had acquired the gene independently. Whilst the rate of tetracycline resistance for South Wales *M*. *hominis* is relatively low, examples of rates between 0 and 5% are not uncommon [65, 66]. Analysis of the MYCO WELL D-ONE showed good concordance with traditional methods of susceptibility testing, with only a minor number of false identification of levofloxacin and moxifloxacin resistance in *Ureaplasma* spp., and clindamycin resistance for *M*. *hominis*. A note of caution needs to be emphasized that the antimicrobial resistance does give much higher false positive rates if not recorded concurrently at the time the species identification wells first turn red. However, with the differential time to positivity for the two separate bacterial species (Fig. 6), this can become quite complicated. Irrespective, it is always important that any putative resistant results should always have an accurate MIC determined for confirmation using CLSI compliant methodologies, and any commercial assay should only be used as an initial screening method for controlled epidemiological studies.

For culture-based assays to successfully and concurrently detect genital mycoplasmas and screen for antibiotic resistance, CLSI-compliant bacterial titres are required (< 10^5^CCU/mL) [21]. Accordingly, bacterial titres between sample types were investigated, revealing swabs to consistently produce titres > 10^5^CCU/mL for both *Ureaplasma* spp. and *M*. *hominis*, following resuspension in 10 mL of saline (Fig. 4), which was particularly striking for matched urine/swab sample analysis (Fig. 5). The comparable sensitivities and specificities for the detection of *Ureaplasma* spp. and *M*. *hominis* (Tables 1 and 2) between sample types coupled with the differences in bacterial load suggest that urine samples are the preferred option for concurrent cultured-based detection and CLSI-complaint AST of genital mycoplasmas.

Whilst the designation of *Ureaplasma* spp. and *M*. *hominis* as pathogen, commensal or pathobiont remains variable, tools that facilitate their identification may aid in resolving such controversy. The MYCO WELL D-ONE displays high sensitivity and specificity values for the detection of *Ureaplasma* spp., with the results being comparable to the widely-used Abbott real-time PCR for the detection of *C. trachomatis* (sensitivity, 92,4%; specificity 99.2%) [67]. Though MYCOWELL D-ONE *M*. *hominis* sensitivity decreased slightly; this is seemingly a universal difference in the ability of culture-based methods to recover these organisms. This does not negate the effectiveness of this assay to detect *M*. *hominis* infection, evidenced by the high values seen across specificity, PPV, NPV and accuracy. Whilst qPCR remains the preferred detection method due to its increased sensitivity and ability to speciate *Ureaplasma* infections, the current European guidelines state routine screening for these organisms is undesirable in lieu of substantiating that the treatment of asymptomatic genital mycoplasma infection is beneficial. Therefore, the sample numbers required to facilitate economically viable high-throughput molecular testing for genital mycoplasmas, as is currently performed for STIs such as *C*. *trachomatis*, are not investigated. In instances of symptomatic non-gonococcal urogenital pathologies, assays such as the MYCO WELL D-ONE provide a simple, economic and accurate method for determining the presence of *Ureaplasma* spp. or *M*. *hominis* in the urogenital tract. Accordingly, it allows clinicians a more comprehensive view of the urogenital microbiome underlying certain pathologies, further elucidating the aetiology of non-gonococcal urethritis, facilitating better-informed guided therapy. This may help to alleviate the concerns of empirical treatments fuelling the generation of multi-drug resistant urogenital pathogens [68] and avoid inappropriate empirical treatment for NGU in cohorts where *Ureaplasma* spp. may be the causative agent [69]. The geographic variability of antimicrobial resistance rates for these organisms highlights the need to continually survey populations for trends in resistance. Whilst the MYCO WELL D-ONE offers a useful presumptive screening method, the need to affirm the result through CLSI-compliant means, or determination of underlying molecular methods of antimicrobial resistance, to prevent the over-reporting of antimicrobial resistance is essential [20, 22].

## Electronic supplementary material

ESM 1 (DOCX 16 kb)

## Data Availability

Available upon reasonable request from the corresponding author.
