# Supplementary material for: MYCO WELL D-ONE detection of Ureaplasma spp. and Mycoplasma hominis in sexual health patients in Wales
Source: Eur J Clin Microbiol Infect Dis. 2020 Jul 28;39(12):2427–40. doi: 10.1007/s10096-020-03993-7 (PMC7669805; doi:10.1007/s10096-020-03993-7)
Supplement: Supplementary file 1 — (DOCX 16 kb) [file 10096_2020_3993_MOESM1_ESM.docx]

**Supplementary table 1 Primers and probes for Mollicutes qPCR multiplex assay**

| **Target** | **Name** | **Sequence (5’-3’)** | **Length (bp)** | **G+C (%)** | **Tm (°C)** | **Amplicon size (bp)** |
| --- | --- | --- | --- | --- | --- | --- |
| *U. parvum* | UUP_FP | AAGGTCAAGGTATGGAAGATCCAA | 24 | 41.7 | 59.3 | 90 |
|  | UUP_RP | TTCCTGTTGCCCCTCAGTCT | 20 | 55.0 | 59.4 |  |
|  | UP_HP | (FAM)-TCCACAAGCTCCAGCAGCAATTTG-(BHQ1) | 24 | 50.0 | 62.7 |  |
| *U. urealyticum* | UUP_FP | AAGGTCAAGGTATGGAAGATCCAA | 24 | 41.7 | 59.3 | 90 |
|  | UUP_RP | TTCCTGTTGCCCCTCAGTCT | 20 | 55.0 | 59.4 |  |
|  | UU_HP | (HEX)-ACCACAAGCACCTGCTACGATTTGTTC-(BHQ1) | 27 | 48.1 | 65 |  |
| *M. hominis* | MH_FP | TCACTAAACCGGGTATTTTCTAACAA | 26 | 34.6 | 58.5 | 94 |
|  | MH_RP | TTGGCATATATTGCGATAGTGCTT | 24 | 37.5 | 57.6 |  |
|  | MH_HP | (ROX)-CTACCAATAATTTTAATATCTGTCGGTATG-(BHQ2) | 30 | 30.0 | 59.9 |  |
|  | | | | | | |
